# Decoding neuroinflammation in Alzheimer’s disease: a multi-omics and AI-driven perspective for precision medicine

**DOI:** 10.3389/fimmu.2025.1616899

**Published:** 2025-06-18

**Authors:** Shiyu Lin, Yijun Zhan, Ruiqi Wang, Jian Pei

**Affiliations:** Department of Acupuncture, Longhua Hospital, Shanghai University of Traditional Chinese Medicine, Shanghai, China

**Keywords:** single-cell RNA sequencing, Alzheimer’s disease (AD), heterogeneity, neuroinflammation, prognostic biomarkers

## Abstract

Alzheimer’s disease (AD) is a common neurodegenerative disease, which is characterized by β-amyloid (Aβ) deposition, Tau hyperphosphorylation, synaptic dysfunction and chronic neuroinflammation. Despite significant advances in research in recent years, effective therapeutic options remain limited. The development of single-cell RNA sequencing (scRNA-seq) has made it possible to analyze cellular heterogeneity in AD brain tissues at high resolution, breaking through the limitation of signal averaging in traditional large-scale tissue analysis. This technology has led to the discovery of novel disease-associated cell subsets, such as pro-inflammatory microglia and reactive astrocytes, and the identification of key molecular markers linked to disease progression. Integrating scRNA-seq with AI-driven analytics and multi-omics platforms further enhances our ability to decode the intricate immune-inflammatory networks underlying AD. This strategy is expected to achieve accurate classification and early diagnosis of AD subtypes, and promote the development of individualized treatment strategies based on individual molecular and immune characteristics.

## 1 Introduction

Alzheimer’s disease (AD) is one of the leading causes of dementia and represents a major public health challenge in the 21st century (1, 2). As a progressive neurodegenerative disease, AD usually manifests as mild memory impairment in the early stage (2), and gradually leads to severe impairment of executive function and cognitive ability as the disease progresses. The pathogenesis of AD involves A variety of complex pathological processes, including β-amyloid (Aβ) deposition, neurofibrillary tangles (NFTs) formation caused by abnormal phosphorylation of tau protein, synaptic dysfunction, blood-brain barrier (BBB) destruction, and chronic neuroinflammation. Abnormal aggregation of Aβ and tau proteins is not only A core pathological feature of AD, but also activates the immune response and induces the formation of inflammasomes, thereby driving the continuous development of neuroinflammation. Mounting evidence implicates immune dysregulation—particularly involving microglia, astrocytes, and infiltrating immune cells—as a central driver of disease progression (3). Yet, the inflammatory response in AD is highly heterogeneous, making it challenging to identify universal therapeutic targets (4, 5). Recent advances in single-cell RNA sequencing (scRNA-seq) (6–8) have enabled high-resolution profiling of cellular diversity and gene expression in AD brains. Unlike bulk RNA-seq, these technologies can resolve rare cell populations and distinguish cellular states, offering new insights into immune–inflammatory interactions (9–11) and lay the groundwork for mechanistic studies and therapeutic innovation (12–14).

## 2 Advances in single-cell sequencing in AD research

**Figure 1:**
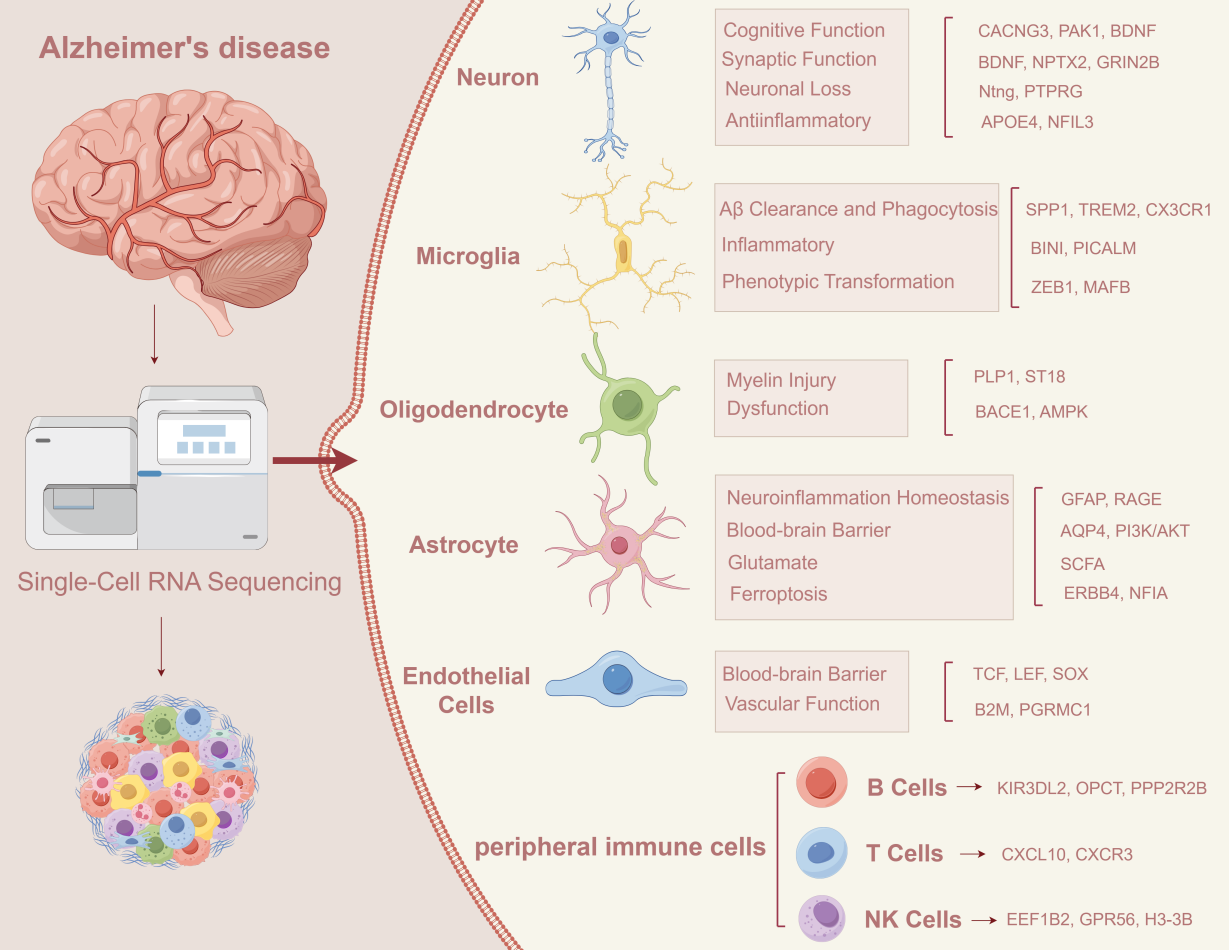
Application of scRNA-seq in AD.

In recent years, the development of scRNA-seq (15–17) and spatial transcriptomics has greatly promoted our understanding of cellular diversity and immune dynamics in AD brain tissue (18, 19). These techniques have revealed specific transcriptional states of microglia, and region-specific subsets of astrocytes and endothelial cells associated with inflammatory signaling and neurovascular dysfunction. Moreover, multi-omics approaches integrating transcriptomics, epigenomics, proteomics, and metabolomics at the single-cell level are uncovering patient-specific molecular signatures that were previously obscured in bulk analyses (20–25). In addition, it identified cell type-specific markers associated with AD, providing new strategies for early diagnosis and targeted therapy (**Figure 1**).

scRNA-seq reveals key transcriptional changes in various cell types in Alzheimer’s disease, including neurons, microglia, astrocytes, oligodendrocytes, vascular endothelial cells and peripheral immune cells, which may jointly drive the occurrence and progression of AD (26). For example, neurons from AD patients show synaptic dysfunction, microglia shift to a proinflammatory state, an imbalance of A1/A2 subtypes of astrocytes (27), myelin damage to oligodendrocytes, and increased BBB permeability. Accompanied by abnormal peripheral immune cell infiltration, these changes may accelerate the process of neurodegeneration (28). Future studies should leverage AI-powered cell lineage tracing (29), spatial multi-omics, and functional genomics to identify critical regulatory nodes and develop personalized, stage-specific interventions for AD.

## 3 Neuroinflammation in AD: cellular and molecular landscape

### 3.1 scRNA-seq reveals inflammation-related cell state transitions

Neuroinflammation plays a crucial role in the progression of AD. With the progression of AD, the inflammatory response is usually aggravated, which is not only derived from the activation of immune cells, but also closely related to neuronal damage, neurodegeneration and loss of synaptic function. Neuroinflammation is triggered by interactions between microglia, astrocytes, endothelial cells, and peripheral immune cells. Neuroinflammation can further exacerbate cellular damage, thereby contributing to disease progression.

In recent years, studies based on AD mouse models (such as 5xFAD, APP/PS1) and human brain tissues have found that a variety of cell types associated with neuroinflammation have characteristic transcriptional patterns (30). Among them, disease-associated microglia (DAM) were first identified by Keren-Shaul et al. in the 5xFAD model, showing Trem2 dependence and up-regulating Apoe, Lpl, Cst7, Itgax and other genes (31). Building on this understanding of microglial function, studies PD-1 deficiency results in increased Aβ deposition, decreased microglia uptake, in APP/PS1 mice, suggesting that dysregulation of the PD-1/PD-L1 axis can exacerbate neuroinflammation and Aβ plaque formation (32). In addition, SLC11A1 was identified as an inflammatory gene associated with AD, and iron overload can induce its expression, suggesting that it may play an important role in iron metabolic-related inflammation (33). T cell infiltration is another key factor in AD neuroinflammation. scRNA-seq studies showed that CD8^+^ T cells activated microglia after entering AD brain tissue, triggering interferon-γ pathway and neuronal damage. CXCL10/CXCR3 axis plays an important regulatory role in this process (34). In conclusion, scRNA-seq can help to clarify the localization of inflammatory cells in brain tissue and its relationship with pathological changes.

### 3.2 Inflammatory signaling pathways and cytokine networks

Neuroinflammation is one of the central features in the pathogenesis of AD, which together with Aβ deposition and abnormal Tau protein drive neuronal dysfunction and neurodegeneration. Neuroinflammation, especially mediated by activated glia, neutrophils, and macrophages, also plays an important role in the pathogenesis of AD.

The occurrence of neuroinflammation is regulated by multiple signaling pathways. TREM2 signaling is critical for microglial metabolic reprogramming, plaque encapsulation, and inflammation regulation. In the CNS, TREM2 is expressed by microglia, and its expression is linked to the immunomodulatory function of APOE (35). NLRP3 inflammasome played a crucial role in AD-related pathology. NLRP3 induced interleukin-18 (IL-18) or interleukin-18 (1L-1B) to further enhance the progression of AD (36). NF-xB was a recognized inflammatory transcription factor that promotes neurodegeneration, was activated in a variety of cell types, and induced transcription of TNF-α, IL-6 and other inflammatory factors (37). The JAK/STAT signaling pathway was one of the key factors promoting neuroinflammation in AD and was affected by the excessive activation of microglia and astrocytes; it suppresses neuroinflammatory responses (38, 39). These inflammatory pathways together constructed a complex network of cytokines, which affect the function of neurons and vascular system.To better understand the role of neuroinflammation in AD, we next focus on cell-type-specific transcriptional changes revealed by scRNA-seq.

## 4 Cell-type-specific investigation of novel biomarkers in AD

### 4.1 Neuronal cells

In terms of cognitive recovery and memory improvement, scRNA-seq has identified key neuronal subtypes in AD and revealed some pathways that may contribute to memory recovery. Hansruedi Mathys et al. showed that cognitive recovery in AD patients was strongly associated with specific subtypes of inhibitory and excitatory neurons. In particular, the expression levels of genes such as CACNG3, PAK1, NPTX2, RPH3A, SVOP, and BDNF in excitatory neurons were positively correlated with overall cognitive function (1). In addition, the BDNF-regulated synapse-associated gene VGF, as well as FGF1 and FGF22 from the fibroblast growth factor (FGF) signaling pathway, were also associated with cognitive function in excitatory neurons (40).

For the regulation of synaptic function, scRNA-seq was used to investigate the role of synaptic and calcium homeostasis related genes in tau-induced neurotoxicity, which was a key factor leading to neuronal loss in AD. Analysis found that a variety of regulators were involved in synaptic function (such as Mctp, Prosap, DIP-t), neuronal excitability (such as Dpp10, GluRIA, Eaat2), and intracellular calcium regulation (such as Trpm, Calx, Cam, RyR) (1, 41).

Regarding the regulation of neuroinflammation, Antara Rao et al. identified two subtypes of proinflammatory microglia with high MHC-II gene expression and highlighted the synergistic role of neuronal APOE, especially APOE4, in AD pathogenesis through interactions with microglia (42). Transcriptome analysis in 5xFAD and trem2^R47H^ mutant models revealed transcriptional changes in microglia and astrocytes that shape microglial and astrocyte inflammatory responses, as well as neuronal activity and BDNF signaling pathway (43). In addition, the up-regulation of NF-κB signaling in inhibitory neurons and the expression of the transcription factor NFIL3 suggest that inflammatory and immune regulatory mechanisms may play a role in AD progression by modulating neuronal function (44). Multi-omics integration is gradually revealing the multi-dimensional regulatory mechanisms of different cell types in AD, and single-cell proteomics and metabolomics can provide functional evidence (45–48).

### 4.2 Microglial cells

In terms of Aβ clearance and phagocytosis, SPP1 was found to be an important factor in regulating microglia-mediated synaptic phagocytosis. In a mouse model of AD, knockout of SPP1 effectively prevented synaptic loss, highlighting its critical role in neurodegeneration (49). Similarly, impaired TREM2 function impaired the ability of microglia to clear Aβ plaques. In contrast, overexpression of TREM2 was able to alleviate pathological changes, showing its potential in AD treatment (50). TREM2 was not only involved in Aβ clearance, but also inhibits excessive neuroinflammation by regulating the phenotype switching of microglia. In addition, disruption of CX3CR1 signaling also reduced Aβ deposition, further underscoring the importance of the microglial receptor pathway in regulating amyloid pathology (31).

Neuroinflammation plays a key role in regulating disease progression. Single-cell analysis revealed several inflammatory response regulators, such as BINI and RELB, which were highly expressed in a variety of microglial states and were involved in the immune activation process (51). In addition, genes such as APOE, BINI and PICALM are considered as risk genes for AD, suggesting their broader regulatory roles in AD pathogenesis. The use of therapeutic antibody AL002c can effectively reduce neurofilament damage and amyloid load, while alleviating inflammatory response, providing a promising intervention (26).

During the progression of AD, the dynamic changes of microglia phenotype reflected their adaptability to pathological signals (52). scRNA-seq analysis has shown that microglia gradually change from a homeostatic state to a DAM, which was dependent on TREM2 signaling pathway, and TREM2 mutations increase the risk of AD (31). One of the DAM subgroups with neuroprotective potential was characterized by up-regulation of Trem2, Tyrobp, Lpl, and Cst7 genes, and down-regulation of P2ry12 and Cx3cr1. In addition, transcription factors such as ZEB1 and MAFB have been found to regulate AD-specific microglial and neuronal transcription patterns, suggesting that cross-type regulatory networks may exist between cells (53).

### 4.3 Oligodendrocytes

In the myelin damage study, Joel W. Blanchard et al. found that promoting cholesterol transport enhanced ApoE4-mediated myelination and improved cognitive function. Studies have shown that APOE4 disrupts cholesterol homeostasis in oligodendrocytes, thereby impairing the ability to generate myelin (54). In addition, activation of Erk1/2 signaling promotes oligodendrocyte (DAO) repair of damaged axonal myelin, ameliorated Aβ-related pathology and cognitive decline in A male APP^NL-G-F^ mouse model (55). Junjie Sun et al. further emphasized that specific marker genes in myelinogenic oligodendrocytes (MOL), such as Mbp, Mobp, Olig2, Mag and Mog, played key roles in myelination, axonal support and signaling (56). Notably, scRNA-seq analysis also identified novel oligodendrocyte markers PLP1 and ST18 (57).

In terms of dysfunction, BACE1 deficiency was found to upregulate the expression of ADAM10, Ano4, ApoE, Il33, and Sort1, which were closely related to Aβ production and clearance. Therefore, targeted inhibition of BACE1 in oligodendrocytes was proposed as a potential strategy to alleviate AD-related Aβ pathology (58). Shiyun Yang et al. demonstrated that overexpression of AK5 in oligodendrocytes could activate AMPK signaling pathway, thereby inhibiting neuroinflammation and apoptosis and promoting oxidative phosphorylation and overall energy metabolism (59). In contrast, elevated PIP4K2A levels may contribute to cellular dysfunction in AD (60). In addition, oligodendrocyte regulatory modules related to APOE and CLU were also identified, providing a new perspective for further understanding the role of these cells in the pathogenesis of AD (61).

### 4.4 Astrocytes

In the regulation of neuroinflammation, astrocytes, especially GfAP-positive subsets (such as AST1 and AST6), play an important role in neuroinflammation, normal aging and a variety of neurological diseases such as AD. These cells act as reactive astrocytes and respond to neuronal injury (62). For example, inhibition of STAT3 signaling in astrocytes can reduce amyloid plaque deposition, improve memory function, and inhibit the activation of proinflammatory factors, thereby slowing the progression of AD (63). Similarly, C5aR1 antagonists were able to suppress glial inflammatory responses, modulate cellular signaling, and prevent cognitive decline (64). In addition, β-amyloid induced the NF-κB pathway by activating RAGE, thereby promoting the inflammatory state, which provides another target for therapy. In AD, reactive astrocytes can release inflammatory factors such as IL-6 and TNF-α and participate in neuronal injury response. Targeting the STAT3 pathway has been shown to slow down neuroinflammation in AD and improve cognitive function (65). In addition, astrocytes also maintained glutamate homeostasis through interactions with neurons, which was essential for reducing neuroinflammation.

Astrocytes also play a key role in maintaining the integrity of the BBB. Studies have found that AQP4 in astrocytes promotes BBB permeability by regulating water transport and assists in the removal of brain waste, thus providing a potential target for AD treatment (66). In addition, studies have revealed that insulin signaling is closely related to BBB function in AD. The interaction between RAGE and the PI3K/AKT pathway is upregulated with age in wild-type mice and may lead to insulin resistance, thereby increasing the risk of AD (67).

Regarding glutamate homeostasis, Yan Sun and his team found that short-chain fatty acids (SCFAs) promote glutamine transport between astrocytes and neurons and alleviate AD symptoms. SCFAs could enhance the communication between astrocytes and neurons, improve glutamate-glutamine circulation, mainly acted on astrocytes to combat nerve oxidative damage, reduce Aβ deposition and Tau protein hyperphosphorylation, and reduced cognitive impairment by remodeling intestinal flora homeostasis (68).

Astrocyte heterogeneity is a key factor in neurodegeneration, where reactive astrocytes can exhibit either a neurotoxic (type A1) or a neuroprotective (type A2) phenotype. Type A1 has neurotoxic characteristics and is induced by activated microglia through TNF-α, IL-1α and C1q. It is manifested as up-regulation of C3, Serping1 and other genes, which can promote neuronal death. However, type A2 upregulates neurotrophins such as S100A10 and PTX3, which are involved in the repair process (69). Specific markers such as GFAP, vimentin, and nestin have emerged as novel therapeutic targets to promote astrocyte-mediated neuroprotection (63). In addition, the pathological down-regulation of ERBB4 and transcription factor NFIA in reactive astrocytes has been found to affect cell-cell interactions, neuronal development and synaptic regulation, thereby aggravating the condition of AD (70). Other studies have shown that NGFR can promote the differentiation of astrocytes into neurogenesis, stimulate cell proliferation and nerve regeneration (71). The continuous expression of NGFR could reduce Aβ plaque and Tau protein phosphorylation, and alleviate the pathological changes of AD. It has also been found that its downstream co-regulators such as PFKP can enhance cell proliferation and neurogenesis (71).

### 4.5 Endothelial cells

In terms of BBB regulation, integrated multi-omics analysis revealed the core regulatory role of TCF/LEF, SOX and ETS transcription factor families in the maintenance of BBB function, providing potential therapeutic targets for nervous system diseases such as AD (72). TCF/LEF, SOX (such as SOX17), and ETS (such as ERG, FLI1) transcription factors maintained the stability of BBB by regulating tight junctions, endothelial differentiation, and anti-inflammatory pathways. In addition, single-cell transcriptomic and immunohistochemical studies showed that IQGAP2, a key molecule that maintains the immune immunity of BBB, was significantly down-regulated in AD. IQGAP2 null mice exhibited a pronounced inflammatory phenotype of brain endothelial cells, as shown- by upregulation of adhesion receptors and antigen presentation related molecules, suggesting impaired BBB function and increased immune cell penetration (73). After the BBB was impaired, cerebrovascular endothelial cells mediated the infiltration and activation of peripheral immune cells through adhesion molecules and chemokines, and acted as antigen-presenting cells to exacerbate neuroinflammation.

In terms of the regulation of vascular function, Shun-Fat Lau et al. observed that endothelial cells in the prefrontal cortex of AD patients showed angiogenic and immune response characteristics. The up-regulation of angiogenic factors and their receptors (such as EGR, FLT1, VWE) and genes related to antigen presentation (such as B2M, HLA-E) suggests that they play an important role in regulating angiogenesis and immune responses in AD pathology (74). In addition, single-cell RNA sequencing results showed that overexpression of PGRMC1 significantly enhanced the proliferation, migration and angiogenesis of endothelial cells, indicating its potential role in regulating the function of cerebrovascular endothelial cells in AD (75).

Studies also found that EIF1 and HSPA1B were key genes closely related to the progression of AD, which were involved in the regulation of peripheral immune and inflammatory responses (76). EIF1 was found to be closely related to cognitive function. Guo et al. integrated single-cell data from ischemic stroke, hemorrhagic stroke, and AD models, and identified Lef1, Elk3, and Fosl1 as potential upstream transcription factors involved in metabolic regulation, suggesting their potential value for therapeutic intervention (77). In addition, the expression of CR1, which was necessary for the clearance of immune complexes, is significantly down-regulated in cerebrovascular endothelial cells of AD patients, suggesting that dysfunction of immune surveillance exacerbated the imbalance of immune homeostasis in the neurovascular unit (78).

### 4.6 Peripheral immune cells

In AD, B cells exhibit significant transcriptomic alterations. The analysis of differentially expressed genes showed that the expression of KIR3DL2, OPCT and PPP2R2B was up-regulated, while FRAT2, WWC3 and SPG20 were down-regulated, and these genes were closely related to the neurodegenerative process (19). scRNA-seq further identified a novel B-cell phenotype with high expression of CD45, enhanced phagocytosis and chemotaxis, and the release of multiple chemokines to recruit peripheral immune cells through the CCL signaling pathway. This phenotypic change may be driven by up-regulation of myeloid-associated transcription factors such as the CEBP family and down-regulation of lymphoid transcription factors such as Pax5 (79).

On the T cell side, the CXCL10-CXCR3 axis played a key role in mediating T cell infiltration and neuronal injury. In particular, the infiltration of CD8^+^ T cells promoted the activation of microglia and further aggravates neuroinflammation and neurodegeneration (34). In addition, cis-regulatory elements co-accessible with the CXCR3 promoter in peripheral CD8^+^ T cells identified, suggesting an epigenetic mechanism associated with AD susceptibility (80). The infiltration of T cells, especially CD8^+^ T cells, activated microglia and further worsens neuroinflammation and neurodegeneration.

Natural killer (NK) cells also play an important role in the pathogenesis of AD. One study identified 17 marker genes associated with AD (such as EEF1B2, GPR56, H3-3B, ZEB2), which may affect immune cell infiltration (81). Through cell communication analysis, researcher identified NK cell subset modules related to AD, and the characteristic genes included RPLP2, RPSA and RPL18A. One specific subgroup was characterized by upregulation of CX3CR1, TBX21, MYOM2, DUSP1, and ZFP36L2, and was negatively correlated with cognitive function in AD patients (82). Moreover, NK cells interacted with other immune cells, such as dendritic cells and macrop-hages, to shape the immune landscape. Activated NK cells induced dendritic cell maturation or apoptosis, thereby indirectly regulating T cell priming (83). In addition, peripheral NK cells infiltrated the brain tissue and regulate the transcription of immune response genes by activating STAT3 signaling pathway, further amplifying the neuroinflammatory response (84).

## 5 Conclusion

Using scRNA-seq, researchers have uncovered transcriptional changes in neurons, glial, and immune cells in AD, elucidating mechanisms of neuroinflammation, synaptic damage, and neurodegeneration. These insights lay a foundation for precision diagnosis and targeted therapy. Integrating AI with single-cell multi-omics and spatial transcriptomics may enable early biomarker discovery and AD subtype identification, facilitating personalized treatment.
